# Roles of eIF5A in the immunosurveillance of cellular senescence

**DOI:** 10.20892/j.issn.2095-3941.2022.0408

**Published:** 2022-10-20

**Authors:** Xiangli Jiang, Fabricio Loayza-Puch

**Affiliations:** 1 Department of Thoracic Oncology, Tianjin Medical University Cancer Institute & Hospital, National Clinical Research Center for Cancer, Key Laboratory of Cancer Prevention and Therapy, Tianjin, Tianjin’s Clinical Research Center for Cancer, Tianjin 300060, China; 2 Translational Control and Metabolism, German Cancer Research Center (DKFZ), Heidelberg 69120, Germany

Senescence is a cellular stress response program that prevents the proliferation of oncogenically activated, genetically unstable, and/or damaged cells. Senescence was first described in 1961, when normal cultured human fibroblasts were found to enter irreversible growth arrest after multiple cell divisions, in a process known as replicative senescence^1^. Several types of senescence have since been identified, such as stress-induced senescence, oncogene-induced senescence, and virus-induced senescence. Despite having distinct features due to their different triggers, senescent cells share several key features. Generally, they have an enlarged and flattened morphology. In response to cellular stresses, they enter a stable cell cycle arrest resulting from the activation of p53–p21 or p16^INK4A^, crucial mediators of cell cycle arrest pathways. Moreover, all types of senescent cells produce a dynamic and bioactive secretome called the senescence-associated secretory phenotype (SASP)^2^. The SASP includes proinflammatory cytokines, chemokines, matrix metalloproteinases, bioactive lipids, vesicles, and growth factors.

Although senescence is considered a protective mechanism against tumorigenesis in young organisms, the accumulation of senescent cells in tissues of older organisms is detrimental, owing to the formation of a proinflammatory microenvironment. Thus, developing strategies to selectively eliminate senescent cells has been a matter of great interest in recent years. Immunosurveillance *in vivo* is one of the most important processes to eliminate premalignant and malignant cells undergoing senescence. This process is mediated by SASP components and by the direct interaction between immune and senescent cells^3^.

The eukaryotic translation initiation factor 5A (eIF5A) is a ubiquitous and highly conserved protein. eIF5A is overexpressed in multiple types of tumors, and its expression is negatively correlated with survival^4^. Originally, it was characterized as a translation initiation factor, but recent studies have indicated that eIF5A promotes ribosome elongation at polyproline or other specific tripeptide motifs^5,6^. eIF5A is involved in multiple molecular functions such as cell proliferation, differentiation, and apoptosis. Recent data suggest that eIF5A plays an important role in cellular senescence, potentially through affecting the expression of SASP components, as well as functions of the immune and vascular systems.

## Potential role of eIF5A in translational control of the SASP

eIF5A has a unique post-translational modification known as hypusination, a process in which the enzymes deoxyhypusine synthase (DHPS) and deoxyhypusine hydroxylase (DOHH) convert a lysine residue at the N-terminus of eIF5A to the unusual amino acid hypusine^4^. The polyamine spermidine is the only substrate for hypusination. To date, eIF5A is the only known protein containing hypusine, which is crucial for eIF5A function^4^.

The p53-p21 axis is a key signaling pathway activated at the onset of senescence^7^. Although p53 is not required to initiate the SASP, it can either promote secretion of several factors or restrain the SASP. eIF5A functions collaboratively in regulating p53 activity^8^. In a UV-irradiated cellular model, Martella et al.^9^ have shown that exposure to UV light increases the binding of hypusinated eIF5A to ribosomes, thereby promoting the translation of p53. Enhanced p53 transcription factor activity further increases the expression of p21. Interestingly, a recent study has revealed a major role of p21 in establishing the SASP. p21 activates retinoblastoma protein (Rb)-dependent transcription and produces a complex secretome^10^. In contrast, our unpublished data have suggested that activation of p53 during cellular senescence stimulates the hypusination of eIF5A by transcriptionally regulating genes involved in the polyamine pathway. Collectively, the interplay between p53 and hypusinated eIF5A may play a role in modulating SASP components.

Expression of the SASP is controlled by inflammasome-mediated IL-1a signaling^11^. Mechanistic target of rapamycin (mTOR) promotes the translation of IL-1a, nuclear factor-κB (NF-κB) activation^12^, and MAPKAPK2 levels during senescence^13^. In yeast, TOR signaling inhibition by rapamycin leads to downregulation of the mRNA and protein levels of the 2 eIF5A isoforms (TIF51A and TIF51B)^14^. eIF5A expression is regulated by the TORC1 pathway under conditions of abundant nutrients; however, whether eIF5A is involved in regulation of the SASP in an mTOR-dependent or-independent fashion during senescence is unknown.

Although experiments in cultured cells have suggested a decreased rate of protein synthesis in senescent cells^15^, production of the SASP requires increased transcription, stabilization, and translation of mRNAs encoding secretory factors. Mechanisms specifically promoting protein synthesis of the SASP during senescence remain poorly understood. One possible explanation for the increased production of SASP factors during times of translational repression is that SASP components might be preferentially translated. Codon choice may be among the mechanisms underlying selective translation. Several studies have shown that eIF5A facilitates translational elongation of stretches of consecutive prolines^5^ and many non-polyproline specific motifs^6^.

## Functions of eIF5A in immune cells and vascular senescence

Senescent cells are subjected to immunosurveillance by multiple components of innate and adaptive immunity. SASP factors attract distinct subsets of immune cells, including natural killer (NK) cells, neutrophils, dendritic cells, monocytes/macrophages, B cells, and T cells. Among them, NK cells, T cells, and macrophages physically interact with senescent cells in pathological and physiological conditions. In a mosaic mouse model of liver carcinoma, reactivation of p53 in p53-deficient tumors induces a cellular senescence program that triggers a potent innate immune response removing tumor cells *in vivo*. The recruited immune effectors include neutrophils, NK cells, and macrophages in the liver^16^. In contrast, hepatic expression of NRas (G12V) elicits oncogene-induced senescence in hepatocytes. In this context, Ras-specific T helper 1 (Th1) lymphocytes have been detected in mice. CD4+ T cells require monocyte-derived macrophages to execute clearance of pre-malignant senescent hepatocytes, thus suggesting that a combination of innate and adaptive immunity is stimulated^17^.

eIF5A and polyamine metabolism have multifaceted roles in maintaining the functions of various subsets of immune cells. First, polyamine synthesis is a hallmark of T cell activation and proliferation. Recent work from Puleston and colleagues^18^ has elucidated the functional implications of polyamine metabolism in Th cell subset fidelity. Disruption of polyamine synthesis or eIF5A hypusination results in failure of T cells to use the correct subset specification. Mechanistic investigations have indicated that a loss of polyamine or hypusinated eIF5A causes profound epigenetic changes driven by histone acetylation and alterations in mitochondrial activity. Second, eIF5A hypusination, catalyzed by DHPS, is a feature of macrophages that reside in the adipose tissue of obese mice. DHPS supports the production of proteins that promote NF-κB signaling in macrophages. Moreover, it stimulates the expression of mRNAs involved in promoting a proinflammatory M1-like state^19^. In contrast, Puleston et al.^20^ have demonstrated that hypusinated eIF5A maintains tricarboxylic acid cycle and ETC integrity in macrophages by modulating mitochondrial protein expression. Inhibition of the polyamine-eIF5A-hypusine pathway blocks mitochondrial oxidative phosphorylation-dependent alternative activation of macrophages.

**Figure 1:**
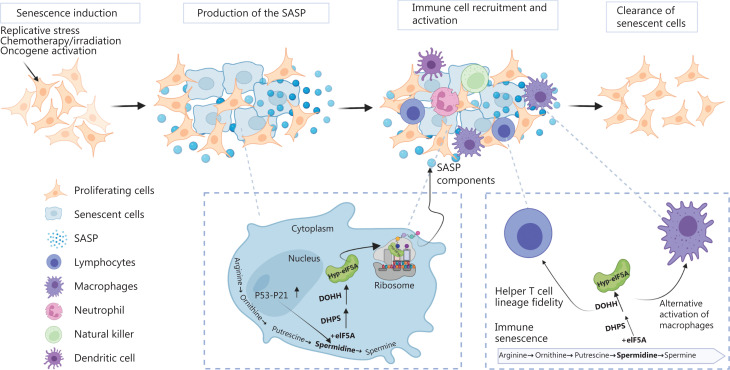
Scheme of the immunosurveillance of senescent cells. Cells enter senescence in response to stresses and begin to produce the SASP, which plays a key role in recruiting and activating immune cells. Senescent cells are then removed through innate or/and adaptive immunity. eIF5A may not only regulate the synthesis of SASP factors but also affect the activity of immune cells during cellular senescence.

Several immune system cell types also undergo senescence^21,22^. Recent studies have identified a role of eIF5A during immune senescence. Zhang et al.^21^ have reported that hypusinated eIF5A specifically regulates the synthesis of the autophagy transcription factor transcription factor EB (TFEB) in B cells. eIF5A has also been associated with autophagy levels in T cells from aged mice. In addition, eIF5A is activated by Kruppel-like factor 5, an essential transcriptional factor during vascular smooth muscle cell senescence. Depletion of eIF5A may lead to vascular disorders, owing to mitochondrial fission and excessive ROS production^23^. Senescent cells may accumulate in aged and diseased tissues, because of a decline of immune system efficiency or the presence of immune-impairing factors in the tissue microenvironment. Therefore, restoring the ability of the immune system or modulating tissue microenvironment may benefit surveillance of senescence (**Figure 1**).

## Harnessing eIF5A to control senescence

During early stages of senescence, the SASP plays primarily a protective role by stimulating the immune system to clear pre-malignant cells. However, long-term exposure to the SASP is generally considered detrimental because of associations with chronic inflammation, malignant conversion of neighboring cells, and spontaneous cell-cycle re-entry of cancer senescent cells with latent stem-like properties. In this context, selective modulation of specific components of the SASP (senostatics) is a promising approach to treat inflammation-associated diseases and cancer. Although many transcriptional regulators of the SASP have been characterized, targeting the SASP at the transcriptional level presents multiple challenges. For example, transcription factors lack binding pockets and present structural disorder, thus limiting the use of small molecule inhibitors. Investigating regulators of the SASP at the translational level is a rational strategy for senostatics.

mTOR inhibitors can selectively inhibit the SASP and decrease inflammation caused by senescent cells. The SASP includes inflammatory cytokines such as IL6, IL8, and monocyte chemoattractant proteins, which alter tissue environments and attract innate immune cells. mTOR inhibition can compromise senescence and its associated immune surveillance, thus resulting in potentially detrimental effects during early stages of cancer or ageing. In contrast, substantial levels of hypusinated eIF5A in senescent cells maintain the expression of diverse SASP components, which in turn may affect immunosurveillance. Moreover, eIF5A appears to be crucial in controlling T cell fidelity and macrophage activation. Hypusinated eIF5A levels decline with age but can be boosted by dietary spermidine. For example, spermidine supplementation reverses B cell senescence and stimulates autophagy in aged CD8+T cells^22^. This evidence suggests that sustaining the levels of hypusinated eIF5A may be important for reversing immune senescence or retaining the integrity of the immune system. Replenishing spermidine could conceivably contribute to immunosurveillance in these senescent conditions.

Senescent cancer cells accumulate in tissues after genotoxic therapies. Therefore, the following questions arise. 1) What are the biological roles of eIF5A during stress/therapy-induced senescence? 2) How does the eIF5A-regulated SASP differ among senescence types? 3) Does a functional switch from an eIF5A pro-growth translation program to a pro-senescence program exist? The underlying mechanisms remain elusive. Late in cancer, proliferating malignant cells have been speculated to outgrow the senescent cells after genotoxic therapy. The innate and adaptive immunity elicited by the early SASP might be less potent in completely eliminating malignant cells *via* mechanisms of immunosurveillance. As the SASP evolves over time, the late SASP components may even contribute to immune evasion. Excessive expression of eIF5A may not necessarily boost immune cell activity. eIF5A may be assumed to have dominant negative effects on senescence in certain contexts. Thus, inhibition of eIF5A activation or its downstream targets appears to be a rational strategy to target cancer senescent cells. To date, no inhibitor directly targeting eIF5A has been developed. However, inhibiting DHPS or DOHH provides a potent route to regulate eIF5A activity. GC7 is the most specific inhibitor of DHPS. However, owing to its adverse effects, wide clinical use is not considered feasible. For instance, mice treated with GC7 show negative effects in T cell proliferation with a selective decrease in Th1 cells^24^. Tanaka et al.^25^ have conducted synthetic studies and obtained bromobenzothiophene, a new compound that targets DHPS, thus opening the door to novel clinical approaches targeting eIF5A.

## Conclusions

Many gaps remain in the mechanistic understanding of the regulation of senescence-associated translation. eIF5A is an intriguing translation factor that may potentially regulate subsets of SASP factors and may have profound effects on the activity of immune effectors and vascular cells. Exploring its targets among different senescent contexts will be extremely important to provide a deeper understanding of translational control during cellular senescence, and potentially yield new avenues to precisely control senescence in age-associated diseases, including cancer.
